# Epidemiology of Chronic Obstructive Pulmonary Disease (COPD) Comorbidities in Lithuanian National Database: A Cluster Analysis

**DOI:** 10.3390/ijerph19020970

**Published:** 2022-01-15

**Authors:** Elena Jurevičienė, Greta Burneikaitė, Laimis Dambrauskas, Vytautas Kasiulevičius, Edita Kazėnaitė, Rokas Navickas, Roma Puronaitė, Giedrė Smailytė, Žydrūnė Visockienė, Edvardas Danila

**Affiliations:** 1 Faculty of Medicine, Vilnius University, Čiurlionio Str. 21, LT-03101 Vilnius, Lithuania; Greta.Burneikaite@santa.lt (G.B.); Laimis.Dambrauskas@santa.lt (L.D.); Vytautas.Kasiulevicius@mf.vu.lt (V.K.); Edita.Kazenaite@santa.lt (E.K.); Rokas.Navickas@gmail.com (R.N.); Roma.Puronaite@santa.lt (R.P.); Giedre.Smailyte@mf.vu.lt (G.S.); Zydrune.Visockiene@santa.lt (Ž.V.); Edvardas.Danila@santa.lt (E.D.); 2 Vilnius University Hospital, Santaros Klinikos, Santariškių Str. 2, LT-08661 Vilnius, Lithuania; 3 Faculty of Mathematics and Informatics, Institute of Data Science and Digital Technologies, Vilnius University, Naugarduko g. 24, LT-03225 Vilnius, Lithuania

**Keywords:** COPD, comorbidities, multimorbidity, clusters

## Abstract

Various comorbidities and multimorbidity frequently occur in chronic obstructive pulmonary disease (COPD), leading to the overload of health care systems and increased mortality. We aimed to assess the impact of COPD on the probability and clustering of comorbidities. The cross-sectional analysis of the nationwide Lithuanian database was performed based on the entries of the codes of chronic diseases. COPD was defined on the code J44.8 entry and six-month consumption of bronchodilators. Descriptive statistics and odds ratios (ORs) for associations and agglomerative hierarchical clustering were carried out. 321,297 patients aged 40–79 years were included; 4834 of them had COPD. A significantly higher prevalence of cardiovascular diseases (CVD), lung cancer, kidney diseases, and the association of COPD with six-fold higher odds of lung cancer (OR 6.66; *p* < 0.0001), a two-fold of heart failure (OR 2.61; *p* < 0.0001), and CVD (OR 1.83; *p* < 0.0001) was found. Six clusters in COPD males and five in females were pointed out, in patients without COPD—five and four clusters accordingly. The most prevalent cardiovascular cluster had no significant difference according to sex or COPD presence, but a different linkage of dyslipidemia was found. The study raises the need to elaborate adjusted multimorbidity case management and screening tools enabling better outcomes.

## 1. Introduction

Health care systems face the challenge of the aging population and an increasing number of chronic non-communicable diseases per patient. Multimorbidity means a co-occurrence of two or more chronic diseases in the same individual at the same period. The increased prevalence of multimorbidity worldwide leads to the high utilization of limited health care resources, lower quality of life, and higher mortality [1,2,3,4,5,6,7].

Chronic obstructive pulmonary disease (COPD) is one of the most prevalent chronic diseases in the world today, causing high morbidity and mortality in the adult population [8,9]. Both death rates and the prevalence of COPD steeply increase with age. More than three million people died from COPD worldwide in 2015, an increase of 11.6% compared with 1990, despite a decrease in the age-standardized rate of 41.9% [10]. Accelerated lung aging may probably cause COPD. The changes in immune systems with aging may also be associated with COPD pathogenesis [11,12,13]. Multimorbidity is common in COPD patients, increasing with age and low socioeconomic status [7,11,14,15]. Many COPD patients have more than two comorbidities. Therefore, multimorbidity occurs in many of them. Addressing the most prevalent chronic diseases may not necessarily address those that impact essential outcomes [15,16,17]. Comorbidities not only increase the burden that people with COPD suffer but decrease quality of life [18]. COPD should be considered as the pulmonary component of multimorbidity [19].

The presence of COPD itself, as well as other comorbidities themselves, contribute to poor health outcomes in COPD patients [14,20,21,22,23,24,25]. Reported comorbidities of COPD patients include a variety of chronic and acute conditions. The studies of COPD patients reported a higher risk of myocardial infarction, lung cancer, depression, and other psychiatric disorders, metabolic syndrome, hypertension, congestive heart failure, chronic kidney diseases, osteoporosis, and diabetes mellitus [20,24,25,26,27,28,29].

The clustering of the comorbidities in COPD patients remains under-investigated [14,15]. Varying methodologies in defining the inclusion criteria, databases used, different clustering methodologies, and various lists of comorbidities lead to mixed published outcomes. Only the cardiovascular cluster is usually included in most of the studies. Other multimorbidity clusters and their prevalence and impact on mortality could be helpful when planning healthcare interventions targeting patients with the most prevalent chronic obstructive airway diseases and multiple chronic conditions [14,16,17,30,31,32,33].

This nationwide cross-sectional study based on individual data aimed to examine the association and clustering of chronic comorbidities in patients with COPD. Cardiovascular diseases (CVD), such as heart failure, arrhythmia, coronary heart disease, diabetes, lung cancer, kidney diseases, depression, and others, were examined, compared to those with and without COPD in the Lithuanian population with at least one chronic disease condition. The study aimed to assess the impact of chronic obstructive pulmonary disease on the probability and clustering of significant comorbidities identifying the target for future interventions.

## 2. Materials and Methods

### 2.1. Dataset

The National Health Insurance Fund (NHIF) database was established in 1999 to reimburse healthcare institutions for healthcare services and statistical needs. The system was used for the management, storage, exchange, analyzing, and reporting of all the services provided by healthcare institutions. The national database contains demographic data and entries on primary and secondary healthcare services, emergency and hospital admissions, and prescriptions of reimbursed medications for chronic diseases.

An anonymized cross-sectional analysis was conducted as a part of a more extensive study of the burden of chronic diseases in Lithuania using NHIF data covering the period from the 1 January 2012 to the 30 June 2014 [1,34,35,36]. Data exporter software was used to extract patients’ demographic information (age, gender), data on 31 chronic conditions using diagnosis codes of the International Statistical Classification of Diseases and Related Health Problems, Tenth Revision, Australian Modification (ICD-10-AM). Data on entries of primary or secondary health care services provided, hospital admissions, and prescriptions of reimbursed medications were included in the study.

As the manifestation of COPD usually most often starts after 40 years of age, only patients aged 40–79 years were included. Many outliers were found in patients aged 80 and older, and this group was excluded from further analysis.

Study design and population: Individuals with records of utilizing care services for COPD (J44.8 from ICD-10-AMD) from the 1 January 2012 to the 30 June 2014 were used to identify individuals suffering from COPD. Only patients diagnosed with chronic obstructive pulmonary disease by a pulmonologist after spirometry and who had received prescriptions for reimbursed bronchodilators for at least 6 months were included (Chart S1 in Supplementary Materials).

No COPD patients were defined as all patients who did not have COPD-related entry in the NHIF database.

Information on diagnoses of CVD (heart failure, arrhythmia, coronary heart disease), diabetes, lung cancer, kidney diseases, and depression was drawn from the NHIF dataset of chronic conditions. According to the WHO International Classification of Diseases, records included the date of primary or secondary health service, hospital admissions, and diagnoses (ICD10-AM). We used the list of 32 chronic conditions associated with the code of ICD10-AM (Table S1) based on Barnett et al. [34,37]. The definition of the diseases was based on the entry of the code.

Other covariates. Details of gender, age, place of residence were also extracted (Charts S2–S4).

### 2.2. Statistical Methods

Two analyses were carried out to assess the association between chronic obstructive pulmonary disease and comorbidities. Descriptive statistics and odds ratios (ORs) for associations were computed. Continuous variables were expressed as mean and standard deviation (SD), categorical variables as numbers and percentages. For these analyses, all individuals with records of cardiovascular diseases: heart failure (I50), arrhythmia (I44–I49), coronary heart disease (I20, I24, I25), as well as diabetes (E10–E14), lung cancer (C33, C34), kidney diseases (N17–N19), and depression (F31–F39) were included.

First, we assessed the prevalence of these comorbidities in those with and without chronic obstructive pulmonary disease. Differences in disease prevalence were tested with Chi-square tests.

Secondly, a cross-sectional analysis to quantify the relationship between COPD and other chronic comorbidities was performed. Our primary outcome was the relationship between prevalent COPD and a diagnosis of each comorbidity under study. Unadjusted ORs and 95% confidence intervals (CIs) of the associations between the outcome variable and each explanatory variable were estimated using logistic regression. Separate multivariable models were built for each disease to look for confounding or effect modification by gender, age, and place of residence.

For the patients with all records of chronic diseases from Barnett’s list, we performed agglomerative hierarchical clustering with Ward linkage for cross-sectional phenotype identification. The Jaccard coefficient was used as a measure of similarity because of the dichotomous nature of the variables. Clustering of the diseases for COPD patients was performed if at least 5% of patients were found to have a comorbidity, separately for men and women. The clusters were depicted graphically from down to the top, marking the same color as a single cluster.

All statistical analyses were performed using STATA version 11 (StataCorp. 2009. Stata Statistical Software: Release 11.0. College Station, TX, USA), STATISTICA version 10 (StatSoft, Inc Tulsa, OK, USA) and R (version3.6.1). R packages “stat” (procedure “hclust”), “vegan,” “dendextend,” “pheatmap,” “fpc” were used to conduct hierarchical clustering and graphical representation. The significance level was defined as *p* < 0.05.

## 3. Results

The final study group consisted of 321,297 patients aged 40–79 years. A total of 1.5% of them had COPD, with prevalence increasing with age and male gender (69.1% vs. 34.7%), more COPD patients lived in rural areas (35.4% vs. 27.0%). (Table 1).

### 3.1. Prevalence Analysis

A significantly higher prevalence was found in COPD patients for CVD (heart failure, arrhythmia, coronary heart disease), lung cancer, and kidney diseases (Table 2). The difference in COPD and no COPD groups for diabetes and depression was not statistically significant.

### 3.2. Cross-Sectional Analysis

After adjustment for sex, age, and place of residence, the multivariate analysis showed the association of COPD with a six-fold increase in the odds of having had lung cancer (OR 6.66, 95% CI 5.68–7.82; *p* < 0.0001), a two-fold increase in the odds of heart failure (OR 2.61, 95% CI 2.46–2.78; *p* < 0.0001), and CVD (OR 1.83, 95% CI 1.69–1.97; *p* < 0.0001) compared with those without COPD (Table 3). A higher risk for arrhythmias, diabetes, kidney diseases, and depression was also found.

### 3.3. Cluster Analysis

**Figure 1:**
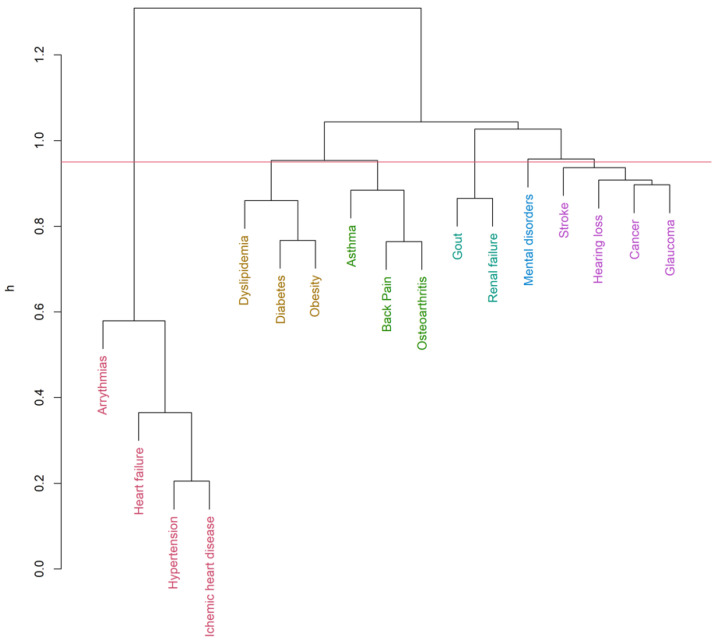
Clusters of comorbidities (COPD group—male, Ward’s method, h = 0.95, average Silhouette width = 0.11, Dunn index = 0.77).

**Figure 2:**
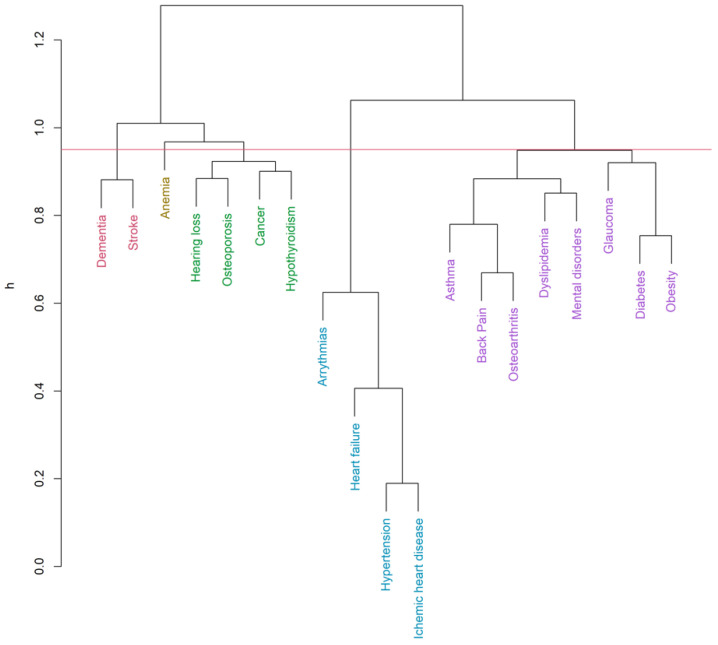
Clusters of comorbidities (COPD group—female, Ward’s method, h = 0.95, average Silhouette width = 0.06, Dunn index = 0.64).

**Figure 3:**
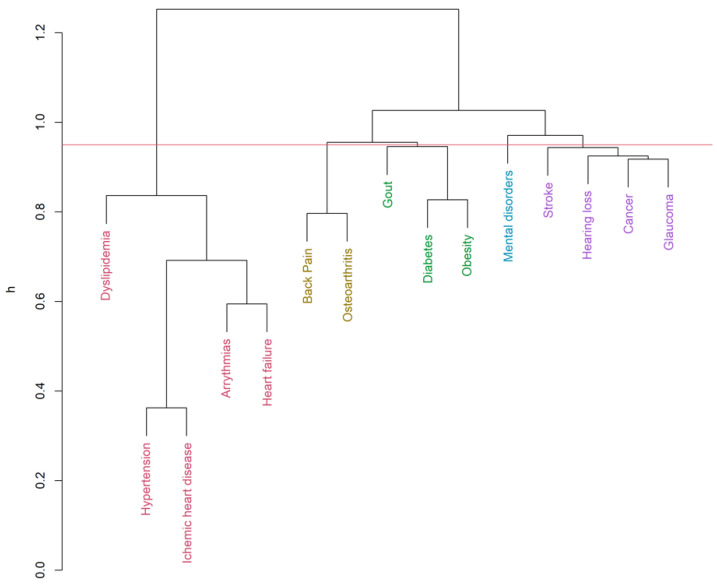
Multimorbidity Clusters (no COPD group—male, Ward’s method, h = 0.95, average Silhouette width = 0.07, Dunn index = 0.75).

**Figure 4:**
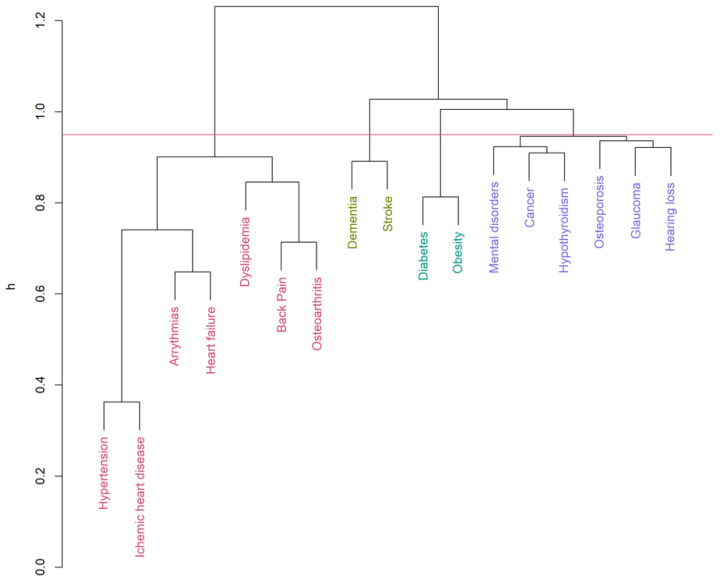
Multimorbidity clusters (no COPD group—female). (Ward’s method, h = 0.95, average Silhouette width = 0.06, Dunn index = 0.73).

Up to 19 diseases from the list of 32 [32,35] were eligible for the clustering inclusion criteria. The hierarchical clustering algorithm (Ward’s method, h = 0.95) identified six clusters for men (Figure 1) and five clusters for women (Figure 2) in the COPD group. Clustering in patients without COPD pointed out five and four clusters accordingly (Figure 3 and Figure 4). The structure of the clusters had some similarities, but a few significant differences were found.

In the male COPD group (Figure 1 and Figure S1, Table 4), a cardiovascular cluster was the most prevalent, with the highest commonness of hypertension and ischemic heart disease. More than half of them were hospitalized at least one time during the study period. Less dominant, but a similar frequency was found in endocrine-metabolic and asthma-musculoskeletal clusters. The prevalence of gout-renal and mental disorders clusters was the lowest, but 70 percent of these patients were hospitalized. A stroke-cancer-sensor cluster was also found. In COPD females (Figure 2 and Figure S2, Table 4), some multimorbidity clusters showed a similar frequency, but the clustering patterns were found to be different. Nevertheless, the most prevalent was a cardiovascular cluster as it was found in men. Asthma-musculoskeletal and endocrine-metabolic clusters were found in COPD males appeared to compose a single cluster, including glaucoma and mental disorders in COPD females. Dementia-stroke cluster was found in females suffering from COPD, but cancer was linked to hypothyroidism, osteoporosis, and hearing loss. The anemia cluster was found only in females with COPD. A total of 70% of females having at least one disease from low prevalent anemia and dementia-stroke clusters had hospitalizations.

Analyzing multimorbidity clusters in males without COPD (Figure 3 and Figure S3, Table 4), dyslipidemia has been linked to the most prevalent cardiovascular cluster. The stroke-cancer-sensor and mental disorders clusters appeared to be the same as in COPD men. Gout was found joining the endocrine-metabolic cluster. A separate musculoskeletal cluster had a high prevalence in men without COPD. In no COPD females (Figure 4 and Figure S4, Table 4), dyslipidemia and musculoskeletal diseases were found to link to the cardiovascular cluster. The dementia-stroke cluster was found in females despite COPD. The endocrine-metabolic cluster consisted only of diabetes and obesity.

The most prevalent cardiovascular cluster (heart failure, coronary heart disease, arterial hypertension, and arrhythmia) appeared to be the same with no significant difference according to sex or COPD presence. Still, hospitalization rates have been higher in the case of COPD presence (Table 4). However, dyslipidemia links to cardiovascular diseases only in patients without chronic obstructive pulmonary disease. In the case of COPD, clustering of dyslipidemia with endocrine-metabolic diseases was found. This finding allows raising a hypothesis of a higher impact of hypoxemia and systemic inflammation in the pathogenesis of CVD in COPD patients rather than dyslipidemia.

Osteoporosis and hypothyroidism were eligible to the clustering criteria only in women despite the presence of COPD. No evidence of asthma in clustering trends of patients without chronic obstructive pulmonary disease was found. Only in men renal failure and gout have met the clustering inclusion criteria, but COPD was significant only for renal failure.

## 4. Discussion

COPD and comorbidities have been studied for many years, but mechanisms of interactions remain unclear [20,24,29]. Airflow limitation, destruction of the lung parenchyma, and systemic manifestations such as systemic inflammation, hypoxia, and hypercapnia are the main features of COPD leading to skeletal muscle wasting, decreased physical activity, osteoporosis, depression, etc. Smoking, hypoxia, and systemic inflammation are the main factors influencing the interactions between COPD and comorbidities [24,38,39,40,41,42,43]. Some pathophysiologic changes in chronic obstructive pulmonary disease can have a direct impact on heart function, causing pulmonary hypertension and heart failure due to right heart overload [15,24,44].

Our research was concentrated on significant and measurable comorbidities, and we used compelling criteria for the COPD definition. As expected, our cross-sectional analysis pointed out an association between having COPD and a diagnosis of CVD. A strong association was found between COPD and heart failure and coronary heart disease, and somewhat weaker for arrhythmia. Clustering of comorbidities also confirmed the significance of CVD in COPD patients. The presence of a higher cardiovascular risk profile in the case of COPD in our cohort corresponds to other studies of general populations [11,24]. The association between COPD and cardiovascular disease, however, is not thoroughly investigated. COPD and CVD share several risk factors, above all smoking and aging. However, individuals with COPD have a 2–3-fold increased risk of CVD compared to controls when adjusted to age and tobacco smoking [45]. Therefore, systemic inflammatory changes caused by COPD are also a risk factor for cardiovascular disease [11,46,47]. Studies of the Swedish and German populations showed similar results. Furthermore, in the Swedish study, patients with co-existing chronic obstructive pulmonary disease and heart failure were reported as having more other comorbidities, such as hypertension, atrial fibrillation, and ischemic heart disease [29,44].

The severity of COPD does not influence the appearance of cardiovascular comorbidities, but the presence of CVD may require a comprehensive therapeutic approach. The consequences of CVD in COPD patients very often are undiagnosed and untreated. The correlation of comorbidities and the severity of bronchoconstriction was not found [29]. There were no data on the severity of COPD in the Lithuanian NHIF database. Still, the selection of patients based on the usage of bronchodilators at least six months per year could suggest higher severity of COPD.

Medications used for the treatment of COPD and comorbidities also influence the interactions among the diseases. Bronchodilators may cause tachycardia, hypokalemia, QTc prolongation, peripheral vasodilation, etc., less common with long-acting inhaled bronchodilators [48]. COPD patients used more cardiovascular-related drugs. Extensive population-based analyses have shown that the risk of mortality could be reduced by prescribing beta-blockers for patients with COPD and HF [29,48]. As our COPD cohort was selected based on receiving bronchodilators for at least six months per year, we did not specify whether long-acting or short-acting bronchodilators had been used. The usage of beta-blockers was not assessed in our investigation.

In a large longitudinal study in the United States and Korea, Hyun Lee et al. showed the difference in the profile of comorbidities by ethnicity and race in COPD patients. White non-Hispanic persons had a higher prevalence of dyslipidemia, myocardial infarction, and osteoarthritis, but in black non-Hispanics, asthma, hypertension, stroke, and diabetes were more prevalent [49]. In addition, Westerner’s comorbidity profile was reported in Japanese patients [50]. White non-Hispanics are predominant in the Lithuanian population, and our study showed the highest prevalence of cardiovascular diseases. Cluster analysis revealed the linkage of dyslipidemia to CVD.

We also found substantial evidence of an increased risk of lung cancer diagnosis in patients suffering from COPD compared to those without the disease. Meta-analysis of 21 studies showed pooled 2.79% prevalence and more than six-fold odds of lung cancer in COPD [51]. Lithuanian data showed a higher prevalence of lung cancer but a similar odds ratio. Higher odds of having had lung cancer were found in the German population, but the prevalence was similar to the general population [29].

Epidemiological studies have identified a strong association between COPD and comorbid psychiatric disorders, including anxiety and depression, with the prevalence of depression ranging from 16% to 88.4%. In addition, individuals with COPD have a higher prevalence of ischemic stroke, transient ischemic attack, sleep disorders, dementia, and Parkinson’s disease, increasing with age [22,52,53,54]. There was no statistically significant difference in the diseased Lithuanian population in the prevalence of depression, but COPD patients were more likely to develop depression. Our study showed higher odds of having had depression in these patients. Depression and other mental diseases end up in a single cluster in COPD males. As the mechanisms of depression in COPD are still not fully understood, declining health status, frailty, aging, systemic inflammation, the impact of smoking, and hypoxemia on brain function possibly have a substantial effect on the development of reactive depression [11,24].

Different researches report a higher prevalence of diabetes mellitus in COPD patients than other populations [20,24,29,55]. In our study, COPD was associated with increased odds of diabetes and kidney diseases, but the prevalence of diabetes was not higher than in other diseased Lithuanians. A gout-kidney cluster was found in COPD males.

Several previous clustering studies performed in COPD patients used different lists of chronic conditions. The inclusion criteria were primarily based on the frequency, and the impact on outcomes analyzed in the peer-reviewed English literature. Only a few studies were prospective, with a low number of patients having COPD confirmed by spirometry included [16,50]. However, our study was in line with those using individual retrospective data from large registries [17,30]. Many of the studies reported five clusters of chronic diseases, but clustering profiles had some differences.

Vanfleteren et al., in a prospective study of 255 COPD patients, used self-organizing maps for comorbidities and identified five clusters: less comorbidity, cardiovascular, cachectic, metabolic, and psychological. Higher systemic inflammation has been found in cardiovascular and metabolic clusters [16]. The Lithuanian study identified the same cardiovascular cluster. Our findings of different clustering trends of dyslipidemia in COPD and no COPD groups suggest a higher impact of hypoxemia and systemic inflammation in the pathogenesis of CVD in COPD patients rather than dyslipidemia. Still, the presence of dyslipidemia in our study could be under-estimated due to possible under-reporting as the statins were not reimbursed during the study period. There were some similarities in other clusters.

In a prospective observational study of 445 Japanese subjects with COPD confirmed by spirometry, Chubachi et al. also identified five comorbidity clusters using the same Ward’s hierarchical clustering methodology as in our study [50]. Lithuanian data showed separate cardiovascular and endocrine-metabolic clusters. A single metabolic and cardiovascular cluster and lower prevalence of CVD in Japanese differs from western studies. A few comorbidities cluster corresponding to Vanfleteren’s study was not found in the Lithuanian population. Probably, due to our definition of COPD, as it was mentioned previously, more severe patients were included. Musculoskeletal cluster detected in Lithuanian data was not found in the Japanese study. Somewhat similarities were found in psychological (vs. mental) and malignant clusters. The anemic cluster has been found only in COPD women. Underweight was not included in our list of diseases.

The chronic conditions corresponding to our list of comorbidities were included in Hansen’s et al. study based on individual data from different registries in Denmark. Using 2 step clustering procedure for chronic diseases in COPD patients, they identified three clusters: comorbidities, including heart diseases, less comorbidity, and other comorbidities without heart disease. The Danish study also showed the importance of the clusters, including cardiovascular diseases identifying heavy users of health care systems. The highest rate of hospitalization was observed in patients with heart disease [30]. We used a similar COPD definition, but the diagnostic algorithm, including prescriptions in Danish studies, was used for all comorbidities. Still, this difference did not have a significant influence on our findings. Our findings on hospitalization confirm the importance of CVD in COPD patients. However, Hansen’s et al. study has not provided sufficient data to compare other clusters.

A Spanish study by Carmona-Pírez et al. in the EpiChron cohort based on demographic and clinical information at the patient level showed that multimorbidity affects about 75% of patients suffering from chronic obstructive airway diseases with higher prevalence than in the general population. The patients were stratified by age and gender. The most common conditions were cardiovascular and metabolic diseases as in Lithuanian data; dyslipidemia does not cluster with cardiovascular diseases [17,56]. The Lithuanian data revealed similar clustering of the most prevalent comorbidities. Stroke-cancer-sensor cluster found in Lithuanian COPD males corresponds to Spanish neuro-substance use-malignancy cluster leading to high mortality. However, there are some differences in the clustering of less prevalent diseases. These differences could be due to the different list of illnesses included in the study.

Divo and Celli had summarized the findings of 11 studies on COPD and comorbidities performed mainly using self-reported data or data from administrative databases. Cardiovascular diseases, lung cancer, osteoporosis, depression, interstitial lung diseases, diabetes, and others lead to decreased functional capacity and increased risk of mortality of COPD patients. The screening for these comorbidities is available in many health care settings using primary or secondary prevention and established treatment algorithms [15]. Literature data and these findings indicate the necessity of screening for the significant comorbidities of COPD patients and adjusting management tools.

## 5. Conclusions

This population-based study of real-life data brings additional evidence to understanding of interactions of significant comorbidities and multimorbidity in COPD. It confirmed the highest prevalence of comorbid cardiovascular diseases with increased hospitalization rate and the CVD and endocrine-metabolic cluster evidence. The presence of dyslipidemia in the endocrine-metabolic cluster in COPD patients as compared to the cardiovascular cluster in the general diseased population presume the differences in the pathogenesis of CVD. Prospective studies on the interaction between chronic obstructive pulmonary disease and cardiovascular diseases would bring more clearance to prevent serious adverse events.

More than twofold odds of having heart failure and coronary artery disease, more than sixfold odds of lung cancer, and higher hospitalization rates in COPD patients suggest the target for screening for significant comorbidities and managing multimorbidity.

Further studies using various databases, the development of standardized clustering methodology of comorbidities, and the clusters’ impact on COPD outcomes would increase the understanding of multimorbidity in COPD. Elaborating more adjusted multimorbidity case management tools could decrease mortality and reduce limited health care resource utilization. In addition, validation of the definition of COPD in administrative databases could bring added value for future investigations.

Strengths. The study was performed using a real-world population and capturing and quantifying health service use for different diseases. The use of medical records of primary and secondary health care settings used in routine work should eliminate differential misclassification due to recall or interviewer prejudice. The COPD group of only patients diagnosed by a pulmonologist suggests the spirometry was performed for all patients despite the absence of the spirometry data in the database.

Limitations. The cross-sectional data analysis could be considered a potential limitation; hence conclusions about causality cannot be made. However, this is appropriate in any cross-sectional analysis. Due to the cross-sectional study design, we could not analyze other diseases recognized as potential comorbidities in COPD patients. Our data are based on the contact with health care providers because of chronic illness in a certain period. Hence, we do not know when the diagnosis was first established if it had happened before 2012. The onset of the disease remains unknown because of the delay between the symptoms and the first visit to a health care provider. However, screening the whole Lithuanian population of chronic illnesses and the period of more than two years reduces the probability of significant discrepancies.

Since our cohort was a sample of a study of chronic disease population, the prevalence of chronic conditions may be higher than in other studies. Although we found a significantly higher risk of manifestations of CVD, heart failure, coronary heart disease, lung cancer, diabetes, and kidney diseases in the COPD group, our methodology may have underestimated the overlap between these diseases. The presence of asthma code in COPD patients has been associated with the level of reimbursement of medication. Nevertheless, the COPD-asthma overlap syndrome could be underestimated.

The COPD definition based on the usage of medications at least six months a year presume the inclusion of more severe COPD patients, and the mild COPD could be undervalued. The stratification by age was not performed during the clustering procedure, but the results of prevalence analysis in different diseases correspond with cluster analysis. The lack of smoking data could be considered a potential limitation, but smoking epidemiology in Lithuania suggests most of COPD patients could be heavy smokers.

## Figures and Tables

**Table 1:** Study population characteristics.

Characteristics	COPD	%	No COPD	%
	*N*		*N*	
Age, mean (SD)	67.2 (8.4)		63.6 (10.1)	
Age group				
40–49	126	2.6	32,341	10.2
50–59	850	17.6	76,602	24.2
60–69	1614	33.4	97,566	30.8
70–79	2244	46.4	109,954	34.7
Total	4834	100	316,463	100
Gender				
Males	3338	69.1	129,505	40.9
Females	1496	30.9	186,958	59.1
Total	4834	100	316,463	100
Residence				
Urban	2285	47.3	178,203	56.3
Rural	1711	35.4	85,391	27.0
Not reported	838	17.3	52,869	16.7
Total	4834	100.0	316,463	100.0

Abbreviations: COPD—chronic obstructive pulmonary disease, N—number, SD—standard deviation.

**Table 2:** Prevalence of other comorbidities in COPD patients.

Comorbidity	COPD	%	No COPD	%	*p*-Value
	*N*		*N*		
CVD	4014	83.0	226,954	71.7	<0.001
Coronary heart disease	4044	83.7	204,776	64.7	<0.001
Hearth failure	3147	65.1	115,500	36.5	<0.001
Arrhythmia	2351	48.6	112,400	35.5	<0.001
Diabetes	609	12.6	40,975	12.9	0.472
Depression	364	7.5	23,077	7.3	0.528
Kidney diseases	278	5.8	12,366	3.9	<0.001
Lung cancer	186	3.8	1304	0.4	<0.001

Abbreviations: COPD—chronic obstructive pulmonary disease, CVD—cardiovascular diseases, N—number, SD—standard deviation.

**Table 3:** ORs from multivariate logistic regression analysis of COPD patients.

Comorbidity	OR	95% CI	*p*-Value
Lung cancer	6.67	5.68–7.82	<0.001
CVD	1.83	1.69–1.97	<0.001
Hearth failure	2.61	2.46–2.78	<0.001
Coronary heart disease	2.32	2.14–2.50	<0.001
Arrhythmia	1.47	1.38–1.55	<0.001
Depression	1.5	1.34–1.67	<0.001
Kidney diseases	1.23	1.09–1.39	0.001
Diabetes	1.15	1.05–1.25	0.002

Abbreviations: COPD—chronic obstructive pulmonary disease, CVD—cardiovascular diseases, OR—odds ratios, CI—confidence interval.

**Table 4:** Clusters by gender and COPD status.

Cluster	Diseases	Patients with ≥1 Diseases in the Cluster					Patients with ≥2 Diseases in the Cluster				
		Prevalence in Group *		Age	Hospitalized Patients †		Prevalence in Group *		Age	Hospitalized Patients †	
		*N*	%	Mean (SD)	*N*	%	*N*	%	Mean (SD)	*N*	%
Males, with COPD, *N* = 3338											
1	Ichemic heart disease, Hypertension, Heart failure, Arrythmias	3294	98.7	66.8 (8.4)	1811	55.0	2970	89.0	67.1 (8.3)	1733	58.4
2	Obesity, Dyslipidemia, Diabetes	1416	42.4	64.8 (8.6)	877	61.9	394	11.8	62.2 (8.5)	295	74.9
3	Osteoarthritis, Back Pain, Asthma	1649	49.4	65.7 (8.7)	915	55.5	515	15.4	65.3 (8.9)	291	56.5
4	Renal failure, Gout	393	11.8	68 (8)	278	70.7	53	1.6	69.5 (7.6)	43	81.1
5	Mental disorders	254	7.6	66 (8.9)	172	67.7	-	-	-	-	-
6	Stroke, Hearing loss, Glaucoma, Cancer	1494	44.8	68.6 (7.7)	887	59.4	353	10.6	70.6 (6.8)	225	63.7
Females, with COPD, *N* = 1496											
1	Dementia, Stroke	202	13.5	71.4 (7.1)	141	69.8	24	1.6	73.3 (4.3)	17	70.8
2	Anemia	81	5.4	68.7 (9.6)	57	70.4	-	-	-	-	-
3	Osteoporosis, Hypothyroidism, Hearing loss, Cancer	566	37.8	69.2 (7.8)	289	51.1	157	10.5	71.2 (6.8)	84	53.5
4	Ichemic heart disease, Hypertension, Heart failure, Arrythmias	1488	99.5	68.3 (8.2)	718	48.3	1348	90.1	68.8 (8)	697	51.7
5	Glaucoma, Mental disorders, Osteoarthritis, Back Pain, Asthma, Obesity, Dyslipidemia, Diabetes	1295	86.6	68.1 (8.3)	650	50.2	934	62.4	67.6 (8.4)	500	53.5
Males, without COPD, *N* = 129,505											
1	Dyslipidemia, Ichemic heart disease, Hypertension, Heart failure, Arrythmias	123,675	95.5	61.9 (10.1)	56,094	45.4	97,832	75.5	62.6 (10)	48,972	50.1
2	Osteoarthritis, Back Pain	41,550	32.1	61.3 (9.9)	17,810	42.9	8448	6.5	61.6 (9.4)	3778	44.7
3	Gout, Obesity, Diabetes	47,110	36.4	60.5 (9.8)	19,027	40.4	10,373	8.0	59.2 (9.4)	5097	49.1
4	Mental disorders	7658	5.9	60.5 (10.3)	3577	46.7	-	-	-	-	-
5	Glaucoma, Hearing loss, Cancer, Stroke	47,050	36.3	65.5 (9.2)	24,207	51.4	9204	7.1	68.4 (8.1)	5125	55.7
Females, without COPD, *N* = 186,958											
1	Osteoarthritis, Back Pain, Dyslipidemia, Ichemic heart disease, Hypertension, Heart failure, Arrythmias	183,463	98.1	65.1 (9.7)	66,721	36.4	158,906	85.0	65.7 (9.4)	61,018	38.4
2	Dementia, Stroke	27,683	14.8	68.4 (9)	16,597	60.0	3012	1.6	72.1 (6.6)	2026	67.3
3	Obesity, Diabetes	66,817	35.7	64.4 (9.2)	23,812	35.6	12,501	6.7	62.6 (9.1)	5777	46.2
4	Osteoporosis, Hypothyroidism, Glaucoma, Hearing loss, Cancer, Mental disorders	82,571	44.2	65.7 (9.4)	29,970	36.3	21,789	11.7	66.6 (9)	8462	38.8

* Group with the same gender and COPD status. † Patients hospitalized at least one time in the follow-up period. Abbreviations: COPD—chronic obstructive pulmonary disease, N—number, SD—standard deviation.

## Data Availability

The data that support the findings of this study are available from Lithuania National Health Insurance Fund, but restrictions apply to the availability of these data, which were used under license for the current study, and thus are not publicly available. Data are, however, available from the authors upon reasonable request and with permission of the Lithuania National Health Insurance Fund.

## Supplementary Materials

The following are available online at https://www.mdpi.com/article/10.3390/ijerph19020970/s1, Table S1. The list of chronic diseases associated with ICD-10-AM, Chart S1: The flow diagram summarizing the process of enrolment, Chart S2: Distribution according to age, Chart S3: Distribution according to sex, Chart S4: Distribution according to the place of residence, Figure S1. Multimorbidity clusters (COPD group—males), Figure S2. Multimorbidity clusters (COPD group—females), Figure S3: Multimorbidity clusters (no COPD group—males), Figure S4. Multimorbidity clusters (no COPD group—females).
