# Supplementary material for: Epidemiology of Chronic Obstructive Pulmonary Disease (COPD) Comorbidities in Lithuanian National Database: A Cluster Analysis
Source: Int J Environ Res Public Health. 2022 Jan 15;19(2):970. doi: 10.3390/ijerph19020970 (PMC8775709; doi:10.3390/ijerph19020970)
Supplement: Supplementary file 1 [file ijerph-19-00970-s001.zip › ijerph-1498542-supplementary.pdf]

Table S1. The list of chronic diseases associated with ICD-10-AM.

Anaemia D50
Hypothyrosis E02; E03; E89.0
Diabetes E10.0-E10.9; E11.0-E11.9
Obesity E66
Dyslipidaemia E78
Dementia F00.0-F00.9; G30.0-G30.9; F01.0-F01.9; F02.0-F02.8; F03
Mental disorders F20.0-F20.9; F30.0-F39; F40.00-F40.9; F41.0-F41.9; F42.0-F42.9; F43.0-F43.9
Parkinson’s disease G20
Multiple sclerosis G35
Epilepsy G40.00-G40.91
Sleep apnoea G47.3
Back pain G54.1; G54.4; G55.1; M51
Glaucoma H40-H42
Blindness H53-H54
Hearing loss H90.0-H90.8; H91.0-H91.9
Hypertension I10-I15
Ischaemic heart disease I20-I25
Arrhythmias I44-I49
Heart failure I50.0-I50.9
Intracranial bleeding I61-I62
Stroke I63-I64; I69
Chronic obstructive pulmonary disease J44.0-J44.9; J96
Asthma J45.0-J45.9
Inflammatory bowel disease K50; K51
Psoriasis L40.0-L40.9
Rheumatoid arthritis M05-M06
Gout M10.0-M10.99
Osteoarthritis M15-M19
Systemic lupus erythematosus M32
Osteoporosis M80-M82
Renal failure N18-N19

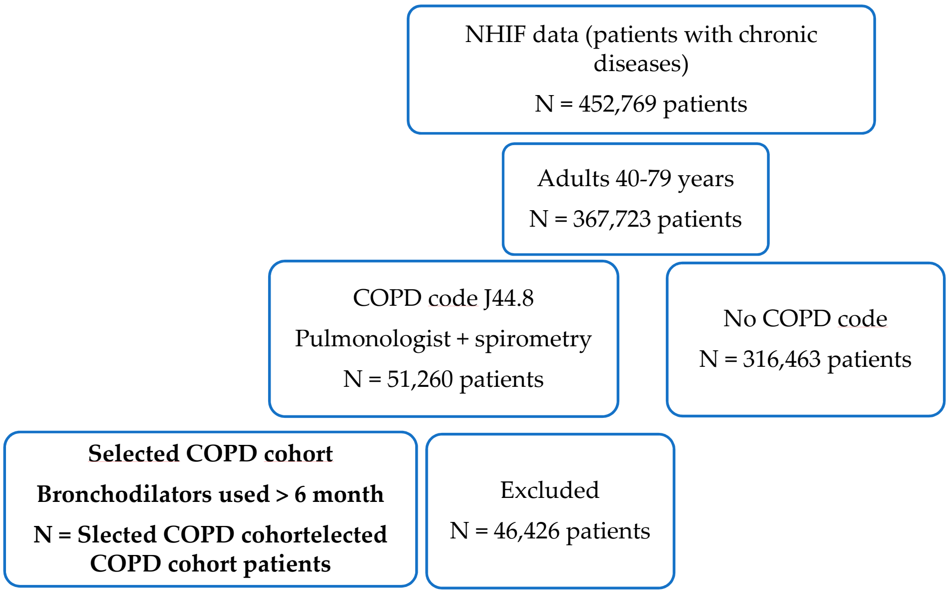

Chart S1: The flow diagram summarizing the process of enrolment.

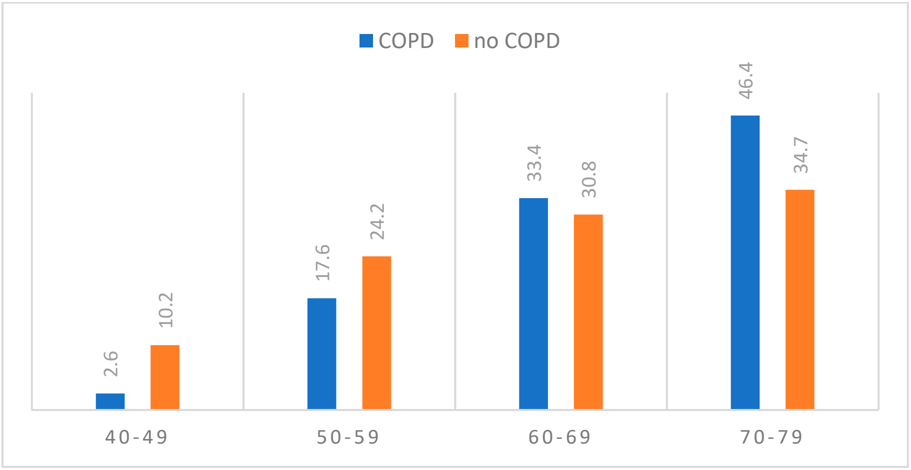

Chart S2: Distribution according to age.

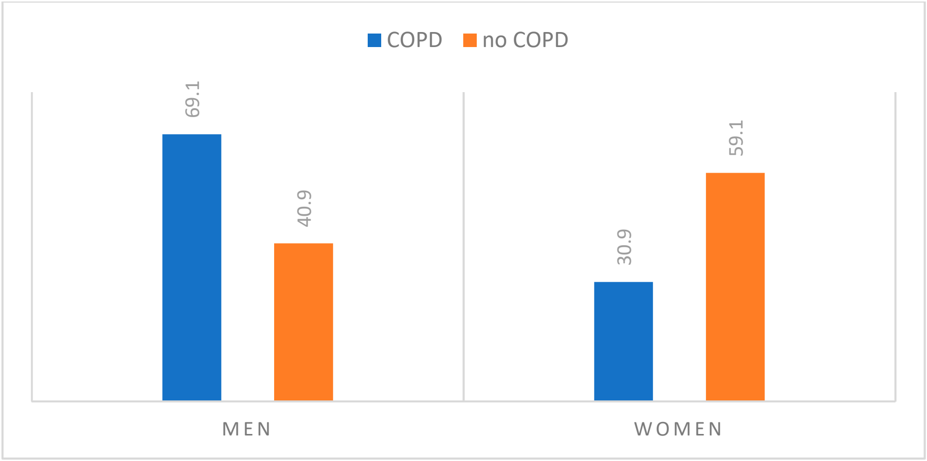

Chart S3: Distribution according to sex.

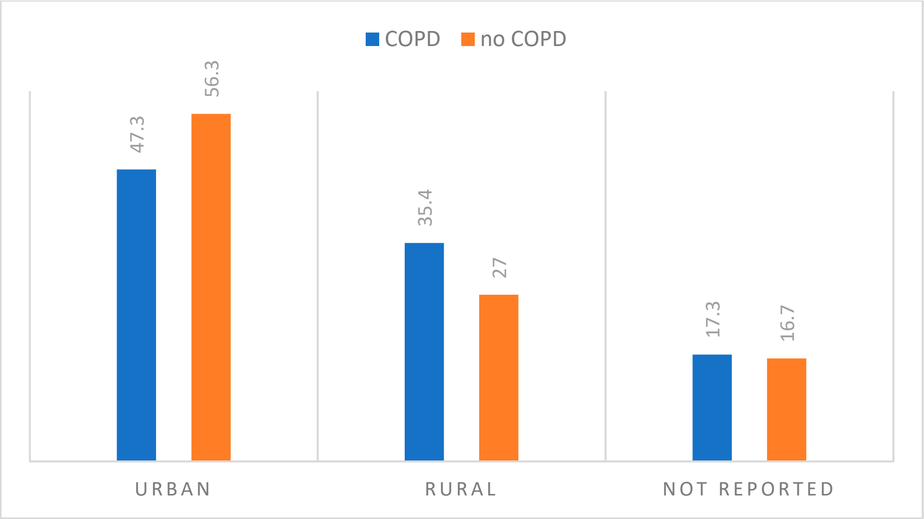

Chart S4: Distribution according to the place of residence.

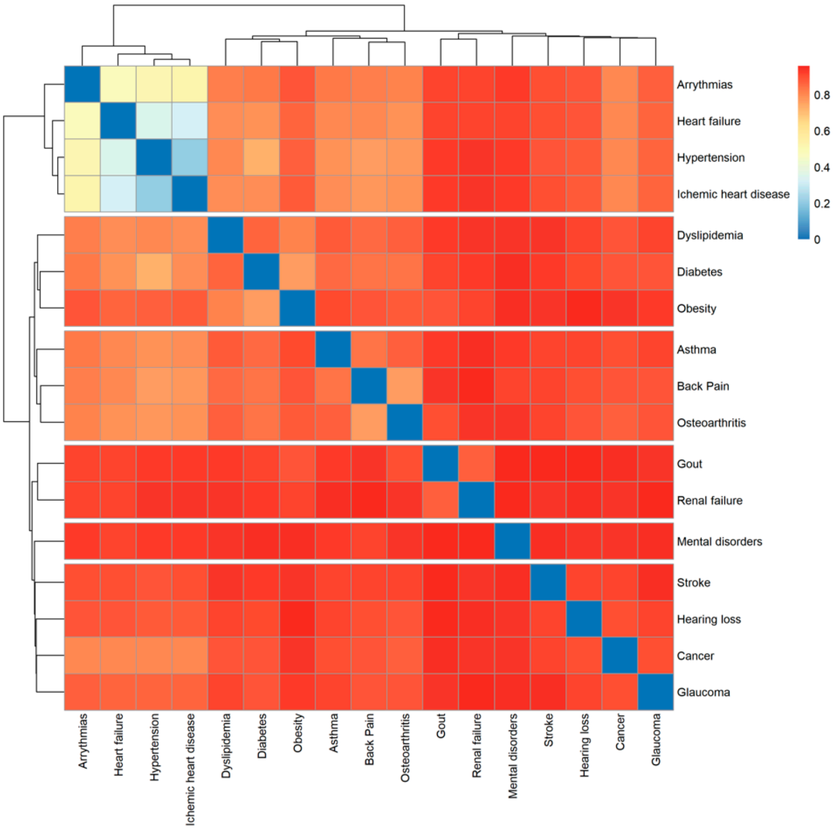

Figure S1. Multimorbidity clusters (COPD group–males).

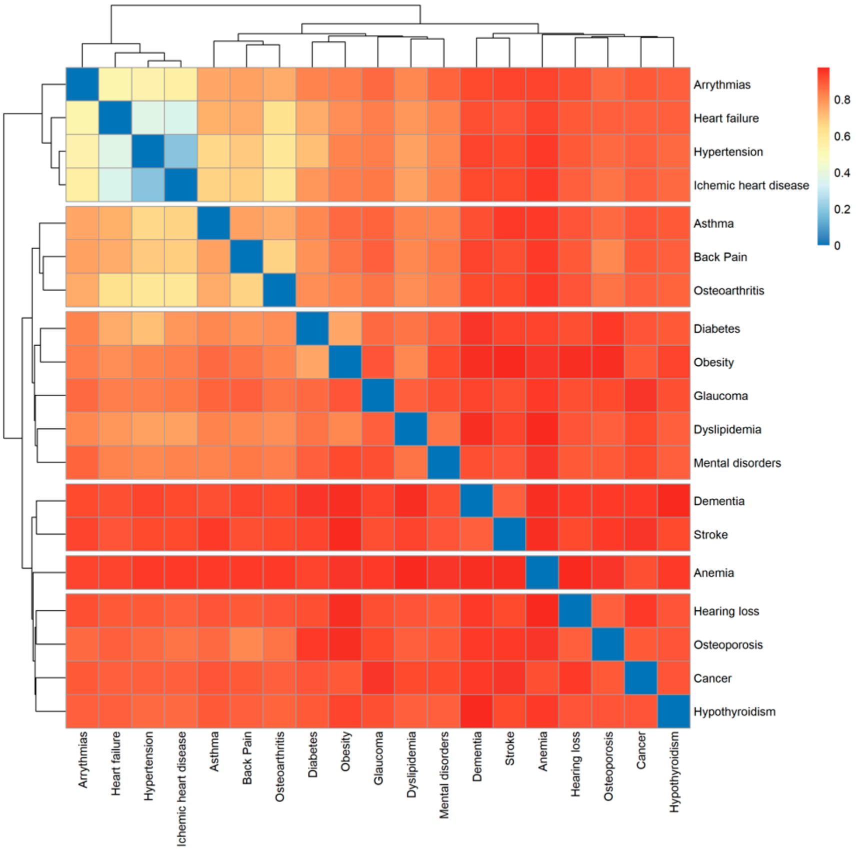

Figure S2. Multimorbidity clusters (COPD group–females).

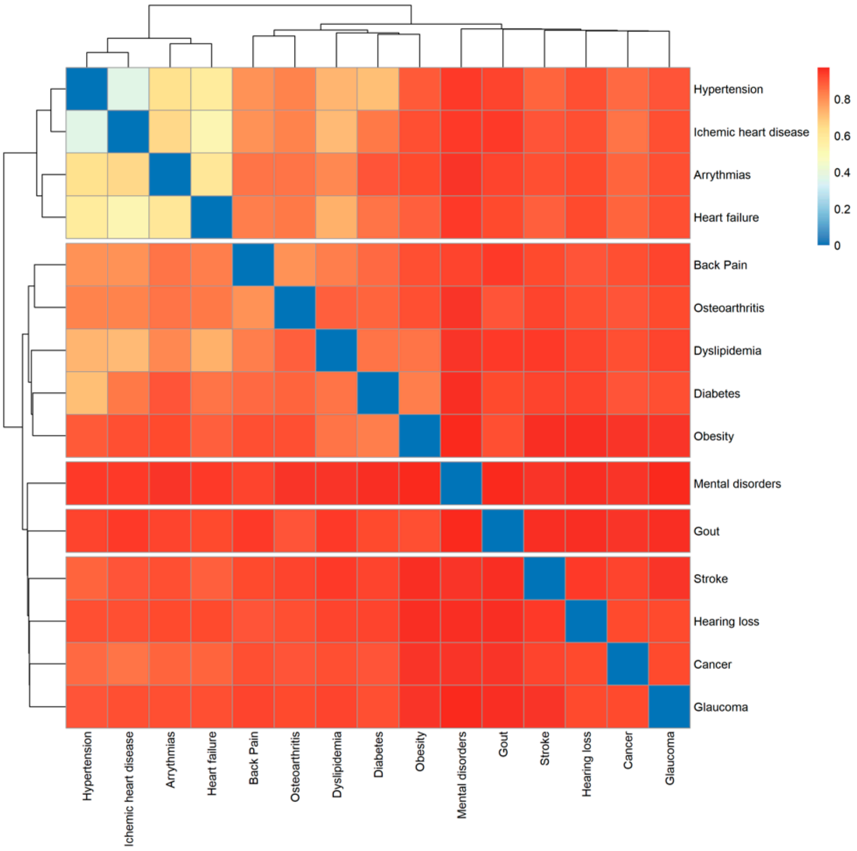

Figure S3: Multimorbidity clusters (no COPD group–males).

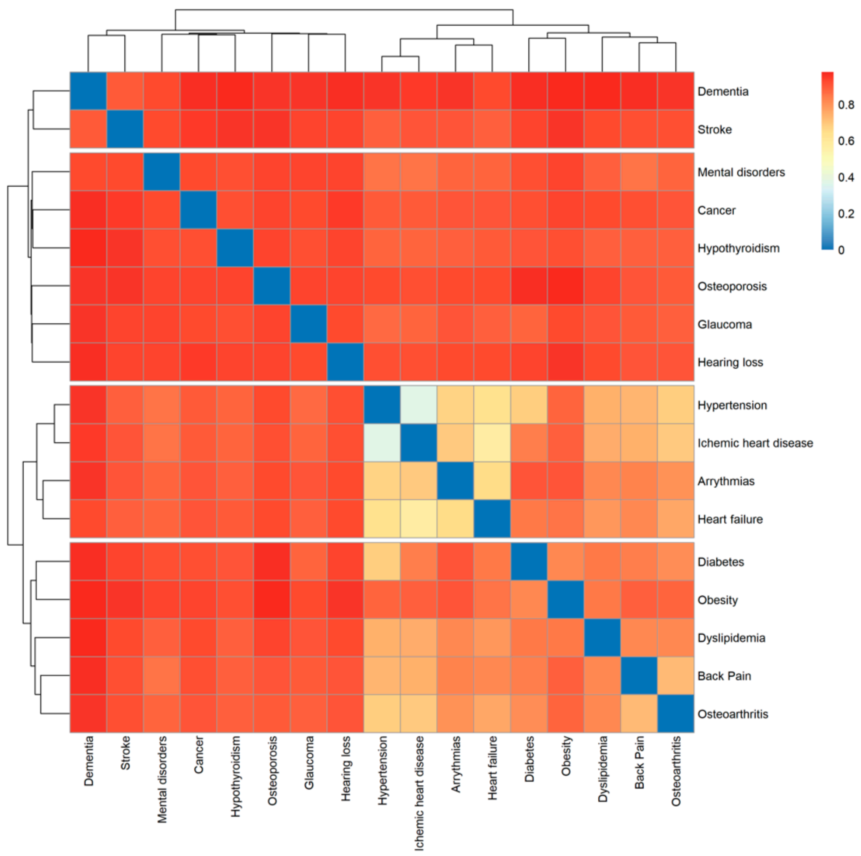

Figure S4. Multimorbidity clusters (no COPD group–females).
